# Auto-segmentation and dose accuracy evaluation of synthetic computed tomography generated from pelvis cone-beam computed tomography images using a cycle-generative adversarial network with a novel loss function

**DOI:** 10.1016/j.phro.2026.100972

**Published:** 2026-04-24

**Authors:** Silambarasan Anbumani, Andrew Keeler, Jiaofeng Xu, Daniel Thill, Nicolette O’Connell, Eric Paulson, Monica E. Shukla, Eenas A. Omari

**Affiliations:** a Department of Radiation Oncology, Medical College of Wisconsin, Milwaukee, WI, USA; b Elekta AB, Stockholm, Sweden

**Keywords:** Adaptive radiotherapy, CBCT, CBCT-based sCT, Synthetic CT, Generative adversarial network, Image quality improvement

## Abstract

**Background and purpose:**

Synthetic CT (sCT) images generated from cone-beam CT (CBCT) images have shown improved image quality over CBCT images. This work evaluated a deep learning (DL)-based sCT model with the potential to facilitate accurate adaptive radiotherapy for pelvis patients.

**Materials and methods:**

A DL-based cycle-Generative Adversarial Network (cycleGAN) model, incorporating a novel loss function to enhance structural image similarity, was used to generate sCT images from CBCT images acquired on a conventional c-arm Linac during intensity-modulated radiotherapy. Auto-segmentation accuracy for 10 pelvic structures was assessed compared to physician-drawn contours using Dice similarity coefficients (DSC), Hausdorff distance (HD), and mean distance-to-agreement (MDA). CT numbers for pelvic organs-of-interest were compared between sCT and planning CT (refCT). Dose, calculated using a Monte Carlo algorithm, was compared using relative dose differences to the gross tumor volume (GTV) for D_98%_, D_50%_, and D_2%_ between sCT and recalculated refCT, alongside 3D-global gamma analysis with 3%/3mm, 2%/2mm, and 1%/2mm criteria.

**Results:**

The sCTs demonstrated high auto-segmentation accuracy with DSC > 0.8 and MDA < 2 mm, except for the small bowel and colon. CT number differences were within published tolerances to achieve less than 1% dose difference excepting structures with substantial gas variations compared to the refCT. The mean dose difference to the GTV was 0.3%±0.6 across D_98%_, D_50%_, and D_2%_. 3D-gamma passing rates were 97 ± 2% for the most restrictive criterion used, 1%/2mm.

**Conclusions:**

The improved cycleGAN-based CBCT-to-sCT model generated high-quality sCT images that may be directly utilized for image segmentation and dose calculation to advance adaptive radiotherapy on conventional Linacs.

## Highlights

• Synthetic image generation was fast, taking less than 1 s per slice.
• Mean computed tomography numbers were within 20 of reference for most soft tissues.
• Auto-segmentation achieved Dice similarity coefficients > 0.8 for most contours.
• Dose calculations were accurate with a 97.4% pass rate for 1%/2mm gamma analysis.

## Graphical abstract

**Figure:**
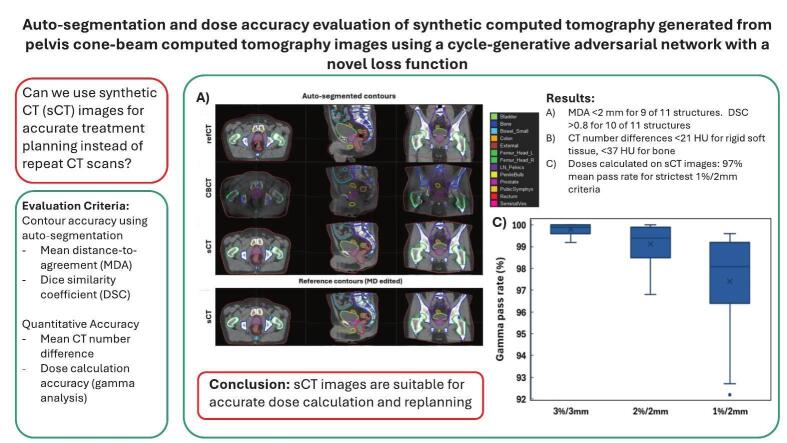

## 1 Introduction

Conventional radiotherapy (RT) planning has traditionally relied on computed tomography (CT) images for tumor and organ-of-interest (OI) delineation and dose calculation. However, promising clinical evidence suggests onboard images can be utilized for adaptive radiotherapy (ART), minimizing radiation toxicities and increasing planning target volume (PTV) coverage [1], [2], [3]. Conventional linear accelerators equipped with cone-beam CT (CBCT) can offer a convenient solution for ART due to wide clinical adoption and relatively low cost. However, CBCT image quality can be adversely affected by increased photon scatter, noise, beam hardening, partial volume effects, and ring artifacts [4], [5] due to the larger field-of-view (FoV) of the cone-beam geometry [6]. These effects pose challenges for auto-segmentation, dose calculation accuracy [7], [8], and quantitative treatment response assessment, which are essential for adaptive treatment planning [2], [9]. Techniques such as deformable image registration (DIR) can be used to compensate for the poor image quality of CBCT by deforming the planning CT to match the daily anatomy [10], [11] However, the inherent uncertainties in DIR can make it difficult to ensure deformation accuracy, particularly for patients with large anatomical deformations [11], [12] such as variable gas accumulation in the bowel, motivating efforts to perform dose calculations directly on the daily CBCT images.

Numerous approaches have been investigated to enhance CBCT image quality, including scatter reduction techniques and measurement-based or software-based scatter correction [6]. Although these methods can mitigate issues such as scatter signals and metal artifacts, achieving sufficient CBCT image quality for replanning remains challenging [13].

The advent of deep-learning (DL) neural network models has had a significant impact on medical image processing and analysis, including image synthesis [14], [15], artifact correction [8], and auto-segmentation [16], [17], [18]. Among DL models for image synthesis, Cycle-Generative Adversarial Network (cycleGAN) models [19] offer several advantages, including the ability to train on both paired and unpaired data, maintain structural integrity, allow for model customization, and produce CT-quality images due to adversarial loss [6], [20]. CycleGAN has shown promise in head and neck and pelvic patient populations [14], [21], [22], [23], [24].

In this study we evaluated a CBCT to synthetic CT (sCT) cycleGAN-based model with a novel loss function for pelvic cancer patients undergoing RT. We aimed to evaluate the use of the CBCT-derived sCT images for online dose recalculation during ART treatments, focusing on auto-segmentation accuracy, CT number consistency, and dose calculation accuracy compared to planning CT (refCT) images.

## 2 Methods

### 2.1 Synthetic CT image generation

A DL-based cycleGAN model was developed and trained using a total of 300 pelvis patients from 9 different institutions and centers [[14], [25], [27], [28]]. Unlike the traditional “unpaired” approach in the original cycleGAN framework, a paired approach was adopted for training based on the paired clinical CBCT and deformably registered CT. The generator architecture was based on the 5-layer 2D ResUnet structure [26], with long skip connections from the Unet architecture adopted for the first three layers across the encoder and decoder and short residual connections adopted from the ResNet architecture. The discriminator network was similar to the original cycleGAN discriminator [19].

**Fig. 1:**
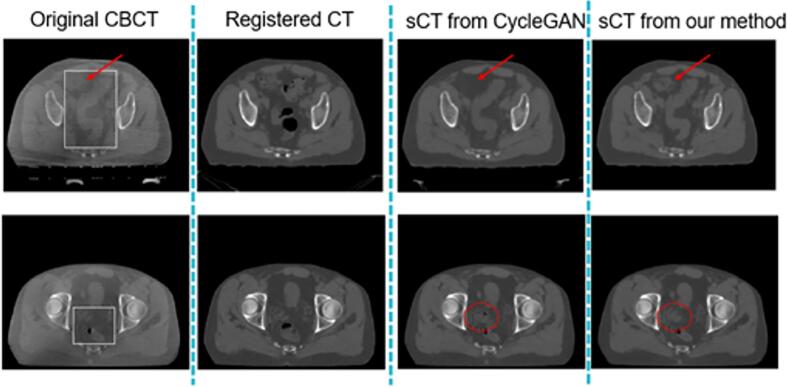
Examples comparing novel structure preservation for conventional cycleGAN and the improved cycleGAN with novel loss functions, both trained on the same datasets with same hyper-parameters. The white boxes indicate areas of structural mismatch between the original CBCT and the deformably registered CT. While the conventional cycleGAN may either remove (top row, red arrows) or produce (bottom row, red circles) structures on the original CBCT, the novel loss function from this work more consistently preserves the CBCT structures. (For interpretation of the references to colour in this figure legend, the reader is referred to the web version of this article.)

Beyond the regular cycle-consistency and adversarial losses, an additional Structural Similarity Index Measure (SSIM)-weighted L1 loss was adopted [[27], [28]]. For a cycleGAN network *G* that transforms CBCT image *x* into sCT image *Pct* and CT image *y* into synthetic CBCT image *Pcbct*, the SSIM-weighted loss L is computed as:

(1) $$L_{{\mathrm{SSIM}}_{\alpha }}\left(G_{cbct2ct},G_{ct2cbct}\right)=E_{x\;p_{\mathrm{cbct}},y\;p_{\mathrm{ct}}}{\mathrm{SSIM}}_{\alpha }\left(x,y\right)∙{\|G_{cbct2ct}\left(x\right)-y\|}_{1}+E_{y\;p_{\mathrm{ct}},x\;p_{\mathrm{cbct}}}{\mathrm{SSIM}}_{\alpha }\left(y,x\right)∙{\|G_{ct2cbct}\left(y\right)-x\|}_{1},$$

where E represents the entire distribution of paired training images. Patch-based SSIM measures between registered images *x* and *y* are computed for each pixel in *x* and *y*. If the local SSIM in a 7x7 pixel patch is above some threshold *α* (*α* = 0.5 in this work), the weights for the L1 norm are set to that SSIM value. Regions with high SSIM thus contribute more to the loss, pushing the model to match the CT image quality. SSIM values below the threshold are set to zero, prioritizing the cycle-consistency loss [[27], [28]] to maintain the original anatomical structures in the CBCT image. This constraint addresses the challenge of misalignment between CT and CBCT images, which cannot always be corrected by DIR. Examples illustrating the improved structure preservation compared to a standard cycleGAN network trained using the same dataset is illustrated in Fig. 1.

Raw CT/CBCT images were preprocessed by resampling the images to 256x256 1.6 mm x1.6 mm pixels and rescaling the pixel intensities from the range [-1000, 2000] HU to [-1, 1]. During postprocessing the rescaling and resampling were reversed to produce images with the original resolution and pixel intensity scale.

The model was developed with Tensorflow 2.0 and trained over 40 epochs. The learning rate was set at 0.0002 for the first 20 epochs and then linearly decreased to zero over the remaining 20. Training used the Adam solver with batch size 4 and was performed on a cluster of 4 A6000 GPUs. The training/validation split of the 300 pelvis images was 90%/10%. The model was deployed for testing in a research version of the ADMIRE tool (v3.48, Elekta AB, Stockholm, Sweden). Inference was executed on an Intel Xeon Gold 6132 Processor (2.6 GHz, 128 GB RAM) with a CUDA Quadro P620 GPU (2 GB memory).

### 2.2 Evaluation dataset

Imaging data from 20 patients undergoing pelvic cancer treatment were collected and retrospectively analyzed under an IRB-approved protocol (PRO00024035) for review of RT images. All patients were treated with intensity-modulated radiotherapy (IMRT) on a conventional C-arm linear accelerator (Infinity or Versa HD, Elekta Instruments, Stockholm) that featured on-board imaging (XVI v5.0.7, Elekta Instruments, Stockholm) between August 2023 and September 2024. Patients with minimal anatomical deviations between the refCT obtained at simulation and the clinical CBCT obtained at the first treatment fraction 7–14 days later were selected for analysis. The patient cohort included eight prostate cancer treatments, ten treatments to the prostate bed and regional lymph nodes for prostate-specific antigen (PSA) recurrence, one rectal cancer treatment, and one bladder cancer treatment.

RefCT images were acquired using a Siemens SOMATOM Drive (Siemens Healthineers, Forchheim, Germany) at 140 kVp with automatic exposure control using CareDose4D. CBCT images were acquired at 120 kVp, 1056 mAs with a 410 x 410 x 264 mm^3^ (“medium”) FoV. Both refCT and CBCT images were reconstructed with a voxel size of 1 mm x 1 mm x 2 mm.

### 2.3 Auto-segmentation performance

Auto-segmented contours for various pelvic structures were generated on the sCT images using deep-learning auto-segmentation (DLAS) software (Contour ProtégéAI v4.0.1, MIM Software, Cleveland, OH). An experienced radiation oncologist adjusted the auto-contours to create reference contours on the sCT following radiation therapy oncology group (RTOG) guidelines [29]. The pelvic structures evaluated included the bladder, colon, small bowel, femurs, lymph nodes, rectum, sacrum, penile bulb, prostate and seminal vesicles (penile bulb, prostate, and seminal vesicle contours were omitted for the one female patient in the study). The autosegmented structures on sCT images were compared with the reference contours using the mean distance to agreement (MDA), Dice similarity coefficient (DSC) and Hausdorff distance (HD) [30]. MDA is defined as the average distance between the closest points on the contours:

(2) $$MDA=\frac{{\vec{d}}_{H,avg}\left(S1,\;\;\;S2\right)\;+\;{\vec{d}}_{H,avg}\left(S2,\;\;\;S1\right)}{2}$$

where S1 and S2 are the surfaces of the auto-segmented structures and the reference contours, respectively, and the directed mean surface distance, denoted as ${\vec{d}}_{H,avg}$, represents the average distance of a point in S1 to its closest neighboring point in S2.

DSC quantifies the volumetric overlap between autosegmented (V1) and reference volumes (V2), providing a standardized metric for segmentation agreement and enabling performance comparisons:

(3) $$DSC=\frac{2|V1\cap V2|}{|V1\left|+|V2\right|}.$$

HD is the maximum distance between closest points on S1 and S2 and indicates the largest segmentation error.

While direct guidance for clinically acceptable agreements between auto-contours and physician-edited contours have not been published, this work bases our contour assessment metrics on the AAPM TG-132 [31] guidance for DIR. We consider tolerances of both DSC > 0.8 and MDA < 2 mm to indicate excellent agreement between the auto-generated and physician-corrected contours.

### 2.4 CT number and dose accuracy evaluation

Mean CT numbers within the physician-corrected sCT structures were compared to the mean CT numbers within the corresponding physician-approved clinical structures on the refCT. For the refCT, the contours were cropped to the treatment FOV. The absolute differences of mean CT number for each structure were compared to published tolerances for various tissues [32], [33], [34], [35]. Spearman’s correlation coefficient [36] was computed to compare CT number line profiles of CBCT and sCT against the refCT profile.

Dose calculation accuracy was assessed by recalculating the plan originally created on the refCT using the MIM SureCalc Monte Carlo dose engine (MIM Software, Cleveland, OH). The dose was recalculated on both the sCT and refCT to remove any effects of different dose calculation algorithms from the comparison. Additionally, considering the limited FOV of CBCT compared to refCT, the refCT volume was cropped to the same FoV as the CBCT for the dose calculation comparison, and beam arrangements were verified to ensure beams remained inside the cropped FOV. This approach eliminated any potential influence of beam arrangement on the results and enabled a consistent and unbiased comparison between modalities. The discrepancies between the refCT and sCT, defined as D_refCT_ – D_sCT_, were assessed by dose-volume histogram (DVH) analysis of the near minimum dose (D_2%_), near maximum dose (D_98%_), and median dose (D_50%_) for gross tumor volume (GTV) contours. The difference in maximum point dose was also recorded. The same conversion curve for CT number to relative electron density (rED) was used for dose calculation on refCT and sCT.

The dose distribution was also compared using 3D global gamma analysis using three sets of criteria: 3%/3mm, 2%/2mm, and 1%/2mm (dose difference/distance-to-agreement). A 10% dose threshold was applied relative to maximum dose of refCT, and a gamma passing rate exceeding 95% was considered acceptable.

## 3 Results

**Fig. 2:**
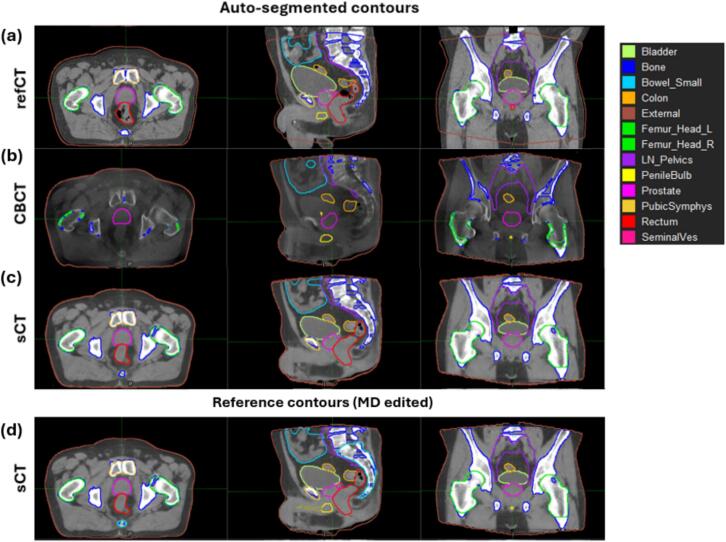
Example axial, sagittal, and coronal views of (a) refCT (Window/Level (W/L): 500/39 HU), (b) clinical CBCT (CBCT) (Window/Level (W/L): 865/16 HU), and (c) sCT (W/L: 500/39 HU) from a representative patient. The auto-segmented contours listed in the legend were generated and overlaid on the corresponding images. (d) sCT with physician-edited contours.

Synthetic CT images generated from a normal size of pelvic CBCT image volume took a few seconds to produce on average with a decent GPU configuration. The sCT images demonstrated substantial improvements in soft-tissue contrast compared to clinical CBCT images, which resulted in substantial auto-segmentation improvements. Particularly, the CBCT image contours were missing key structures such as the bladder and would require substantial editing to be suitable clinically. (Fig. 2).

**Fig. 3:**
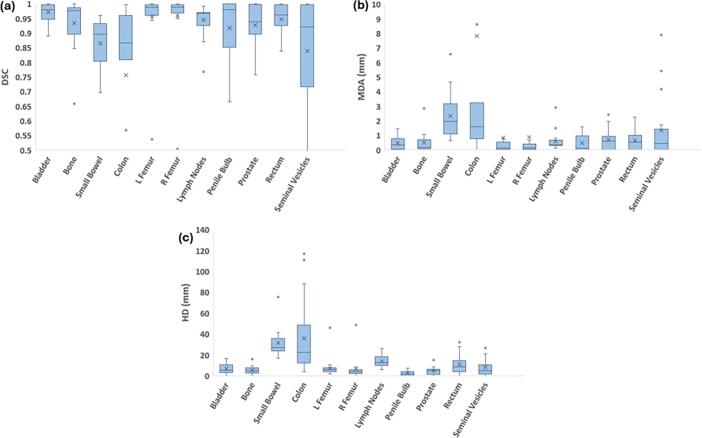
Comparison of (a) DSC, (b) MDA, and (c) HD between auto-segmented and physician-edited contours of pelvic OIs. N = 20 patients (N = 19 patients for the prostate, penile bulb, and seminal vesicles structures).

By contrast, the auto-segmented sCT contours showed good quantitative agreement with the reference contours (Fig. 3). Only the colon and small bowel exceeded our defined tolerance (MDA > 2 mm) due in part to the high complexity of those structures.

**Fig. 4:**
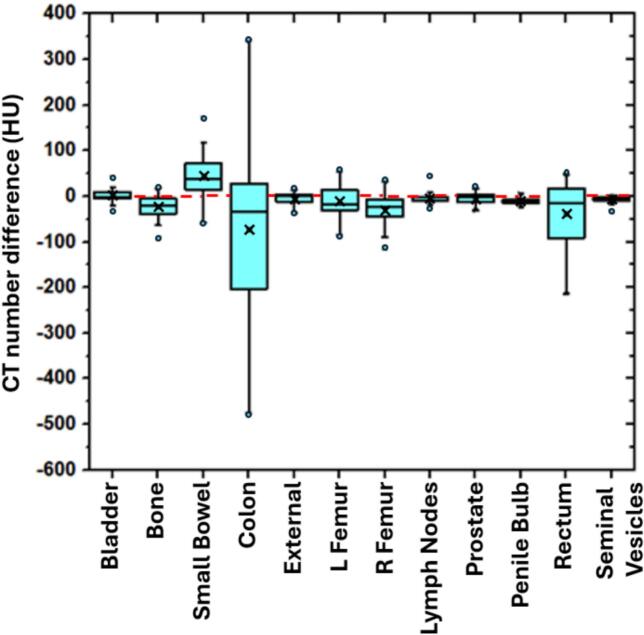
Box-whisker plot of CT number difference (refCT-sCT) differences between sCT and refCT for the pelvic OIs. **x** represents the mean CT number difference for each structure.

The mean CT numbers for the sCT structures also showed good overall agreement to those of the refCT (Fig. 4). The mean absolute differences were as follows: bladder (10.9 HU), bone (25.3 HU), small bowel (49.9 HU), colon (149.2 HU), left femur (29.2 HU), right femur (36.9 HU), lymph nodes (13.7 HU), penile bulb (12.1 HU), prostate (10.4 HU), rectum (59.8 HU), and seminal vesicle (11.2 HU). In certain patients, the presence of gas within the gastrointestinal structures resulted in high variation in CT numbers of the sCT images compared to refCT. However, the proposed cycleGAN-based model was observed to remove some streak artifacts caused by this gas motion in the CBCT images (**Figure S-1)**.

**Fig. 5:**
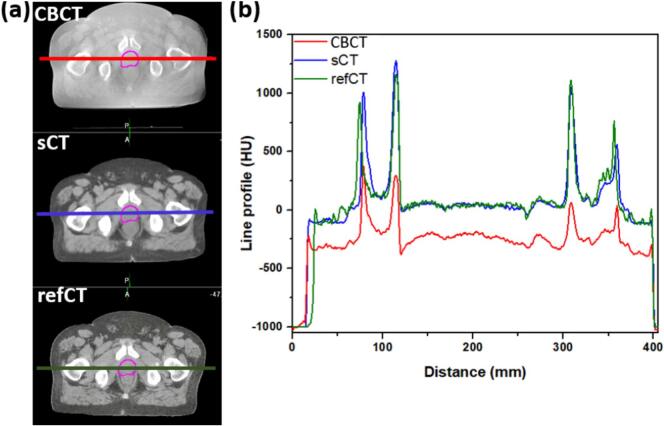
Example CT number line intensity profiles in an axial slice intersecting the femur bone and prostate target for an example patient: (a) CBCT (W/L: 400/-360), (b) sCT (W/L: 500/39), and (c) refCT (W/L: 500/39) images.

CT number line profiles showed improved agreement between refCT and sCT compared with refCT and CBCT (Fig. 5). Spearman’s coefficient of 0.9 for sCT and 0.8 for CBCT indicated that sCT better preserved the shape of CT number profile compared to the large fluctuation along the same anatomical line for the CBCT image.

**Fig. 6:**
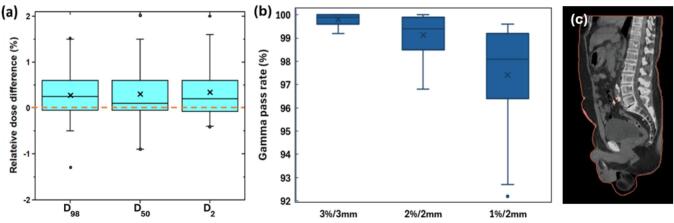
(a) Dose difference of D_98%_, D_50%_, and D_2%_ for the sCT gross tumor volume (GTV) relative to refCT dose, (b) 3D gamma analysis with criteria of 3%/3mm, 2%/2mm, and 1%/2mm, and (c) example CT image showing failing gamma regions.

The largest dose differences to the GTV were below 1.7% of the refCT dose (Fig. 6**a**). The median GTV dose on sCT was slightly higher than on refCT for D_2%_ (0.3%) and D_98%_ (0.2%), while D_50%_ differed by 0.1%. Maximum point dose differences ranged from 0.08% to 1.5% with extreme cases attributable to variations in rectal gas content. 3D gamma analyses using criteria of 3%/3mm, 2%/2mm, and 1%/2mm resulted in passing rates of 99.8 ± 0.2%, 99.1 ± 0.9%, and 97.4 ± 2.3% respectively (Fig. 6**b**). Failing gamma regions were generally areas showing difference between gas and soft tissue, such as the surface of the skin (Fig. 6**c**).

## 4 Discussion

The improved cycleGAN sCT model produced high-quality sCT images from pelvic CBCT images that enabled accurate auto-segmentation and dose calculations. Auto-segmentation of most of the pelvic OI structures showed a high level of consistency (>0.8 DSC and < 2 mm MDA) with reference contours. CT numbers also showed good agreement between sCT and refCT, with mean CT number differences below 20 HU for rigid soft structures and 50 HU for bony anatomy. Recalculated doses also showed high levels of agreement between the refCT and sCT, with mean gamma pass rates of 97.4 ± 2.3% for the most stringent 1%/2mm criteria.

The auto-segmentation performance of pelvic OIs in this work was higher than previously reported for the cycleGAN based sCT images [14]. Eckl et al reported the DSC values of bladder, prostate, rectum, and seminal vesicles are 0.9 ± 0.04, 0.85 ± 0.03, 0.81 ± 0.03, and 0.66 ± 0.08, respectively, using DIR-based contours from refCT to sCT images [14]. In comparison, this study showed improved DSC values of 0.97 ± 0.05, 0.92 ± 0.07, 0.96 ± 0.05, 0.8 ± 0.2, respectively for the same structures. These segmentation results may reflect the combined effect of improved sCT quality and the use of a clinically validated DL-based segmentation model to avoid registration inaccuracies inherent in DIR. However, these results are potentially affected by automation bias, which may provide inflated DSC values compared to *de novo* contouring.

Quantitative evaluation of CT numbers in the sCT images demonstrated good agreement with the refCT, indicating reliable CT number fidelity. CT number difference between sCT and refCT for rigid soft tissue structures (±20 HU), as well as the bony anatomy (±50 HU), fell within the published recommendations [32], [33]. According to Langen et al, for Tomotherapy-based IMRT, CT number deviations should stay within ± 20 HU for soft tissues and within ± 50–80 HU for lung and bone-like materials to keep dose differences within 2% [34], with similar recommendations from the AAPM TG-179 and TG-148 [35]. Our results for the sCT images are generally consistent with these CT number tolerances for various tissue types. However, large CT number differences were observed in the small bowel, colon, and rectum due to the variation in gas quantity in those structures [37].

Excellent dose accuracy was observed on the sCT compared to the refCT, with an average relative dose difference of less than 0.5% for the GTV and an average gamma passing rate of 99.1 ± 0.9% for 2%/2mm criteria. The results are consistent with or improve upon previous cycleGAN studies, which found 2%/2mm gamma pass rates of 98.4% [23] and 97.8% [24] for head-and-neck sites, and 93% [38] for the pelvis.

The evaluated cycleGAN-based DL model leverages paired imaging data to transform artifact-prone CBCT images into high-quality CT-like images, improving consistency in CT number and dose calculation accuracy. Traditional methods for correcting scatter and artifacts in CBCT images, such as iterative scatter correction and noise filtering, can be computationally demanding and only incompletely resolve the issues [6]. Recently, CBCT detector panel designs have resulted in improved image quality [39], [40]; nevertheless, these enhancements can be expensive and resource-intensive [39], [10]. In contrast, sCT offers a solution for utilizing CBCT images with existing hardware.

This work has several limitations. Firstly, the CBCT images in this study were acquired with a medium FOV of 41x41 cm^2^ with minimal artifacts present. In some instances, such as for larger patients, a larger FOV of 51x51 cm^2^ will be required to capture the whole anatomy. Also, patients with metal implants or gas motion during CBCT acquisition show imaging artifacts that would hinder the accuracy of dose calculations. While some artifacts were removed by the proposed cycleGAN-based model (**Figure S-1**), the handling of more severe artifacts remains unevaluated. Secondly, though not observed in this work, hallucination occurrence awareness is necessary in DL models [41] and implementation of a thorough quality assurance program must take this into consideration. Lastly, the analysis in this work is restricted to considering the effects of the sCT image quality on auto-segmentation accuracy and dose calculation accuracy. Future work will assess large FOV models, incorporate more robust artifact and hallucination management, and further evaluate the suitability and impact of sCT images for decision-making in ART workflows.

Overall, this study demonstrated high-quality sCT images can be generated from daily CBCT images by utilizing a modified cycleGAN model [[27], [28]]. The effectiveness of the model was confirmed by improved CT number fidelity and auto-segmentation accuracy. Moreover, quantitative dose evaluations indicate that the sCT images are suitable for accurate dose calculation.

## CRediT authorship contribution statement

**Silambarasan Anbumani:** Writing – original draft, Visualization, Validation, Investigation, Formal analysis. **Andrew Keeler:** Writing – review & editing, Visualization, Data curation. **Jiaofeng Xu:** Writing – review & editing, Software, Methodology. **Daniel Thill:** . **Nicolette O’Connell:** Project administration. **Eric Paulson:** Writing – review & editing, Resources. **Monica E. Shukla:** Writing – review & editing, Visualization, Resources. **Eenas A. Omari:** Writing – review & editing, Visualization, Validation, Supervision, Resources, Project administration, Methodology, Funding acquisition, Conceptualization.

## Declaration of competing interest

The authors declare that they have no known competing financial interests or personal relationships that could have appeared to influence the work reported in this paper.

## Appendix A Supplementary data

Supplementary Data 1 A figure depicting the reduction of minor streak artifacts in sCT images compared with CBCT images.
