# Supplementary material for: Auto-segmentation and dose accuracy evaluation of synthetic computed tomography generated from pelvis cone-beam computed tomography images using a cycle-generative adversarial network with a novel loss function
Source: Phys Imaging Radiat Oncol. 2026 Apr 24;39:100972. doi: 10.1016/j.phro.2026.100972 (PMC13181291; doi:10.1016/j.phro.2026.100972)
Supplement: Supplementary Data 1 — A figure depicting the reduction of minor streak artifacts in sCT images compared with CBCT images. [file mmc1.pdf]

Supplemental Data:

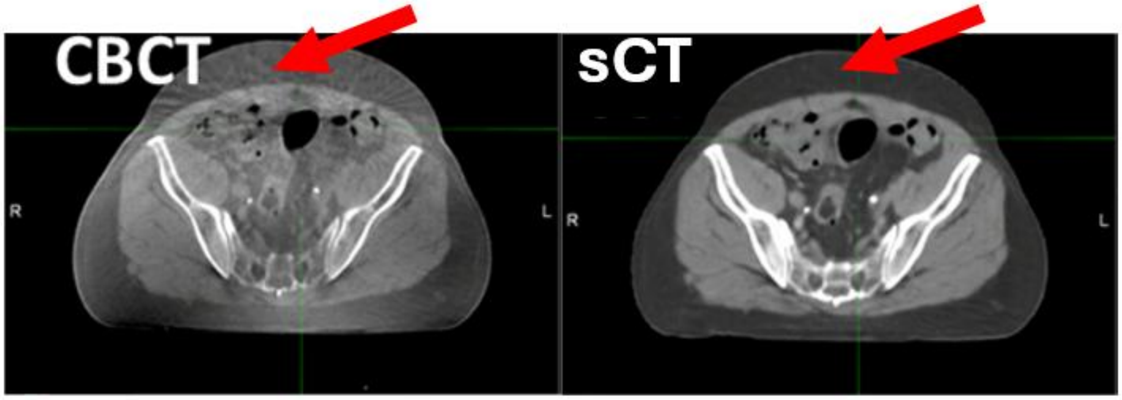

Figure S-1: CBCT showing streaking artifacts caused by the large gas quantity in the bowel region (streaks indicated by red arrow). These streak artifacts were corrected by the DL network in the sCT images.
